# The importance of slaughterhouses in monitoring the occurrence of tail biting in pigs – Review

**DOI:** 10.17221/85/2023-VETMED

**Published:** 2023-09-28

**Authors:** Martin Svoboda, Nikola Hodkovicova, Andrzej Siwicki, Wojciech Szweda

**Affiliations:** ^1^Ruminant and Swine Clinic, Faculty of Veterinary Medicine, University of Veterinary Sciences Brno, Brno, Czech Republic; ^2^Department of Infectious Diseases and Preventive Medicine, Veterinary Research Institute, Brno, Czech Republic; ^3^Department of Microbiology and Clinical Immunology, Faculty of Veterinary Medicine, University of Warmia and Mazury in Olsztyn, Olsztyn, Poland; ^4^Department of Epizootiology, Faculty of Veterinary Medicine, University of Warmia and Mazury in Olsztyn, Olsztyn, Poland

**Keywords:** meat inspection, prevalence, tail lesions, scoring system

## Abstract

Tail biting in pigs represents a very serious problem in modern pig farming, particularly with the intensification of the industry. It is considered a multifactorial syndrome and can be caused by various factors, leading to significant economic losses through reduced weight gain and partial or total condemnation of slaughtered carcasses due to secondary bacterial infections. The aim of this article is to summarise the current knowledge regarding tail biting in pigs, with a primary focus on the use of slaughterhouses for evaluating tail lesions and monitoring their prevalence. The introduction addresses the factors influencing the incidence of tail biting in pig farms and prevention strategies. Subsequent sections cover topics such as tail docking, the negative effects of pig tail biting, the advantages and drawbacks of examining tail lesions in slaughterhouses, and the methodical procedure for evaluating such lesions. Additionally, the article discusses the relationship between tail lesions and meat inspection findings, as well as the prevalence of tail lesions in various European countries.

## INTRODUCTION

Tail biting is considered to be an economic and welfare problem (vom Brocke et al. 2019; Honeck et al. 2019). Tail biting is an undesirable behaviour occurring worldwide, primarily in growing pigs, which raises a welfare concern and can cause severe economic losses (Henry et al. 2021; Gomes et al. 2022). The simplest definition is that tail biting is the oral manipulation of a pig’s tail by another pig (Henry et al. 2021). However, the problem is much more complex, with varied reasons and severity for its development; in general, the aspects of its occurrence are multifactorial (Taylor et al. 2010; Henry et al. 2021).

The main reasons for the tail biting behaviour are connected to the unsatisfying environment (temperature, ventilation, noise, etc.), higher stocking densities, low quality or quantity diet, the presence of diseases, and overall low welfare conditions (Munsterhjelm et al. 2016; Henry et al. 2021; Boyle et al. 2022; Gomes et al. 2022). Generally, the lack of chewing material is one of the main reasons for developing this unpleasant behaviour, which can be addressed by providing straw or other enrichment material (Telkanranta et al. 2014; Gomes et al. 2022).

Three types of tail biting are distinguished (Taylor et al. 2010; Henry et al. 2021), i.e. two-stage type, sudden-forceful and obsessive. The two-stage type is commonly referred to by most authors. According to Taylor et al. (2010), it is described in lying pigs with no or passive reaction of the victim. This type of tail biting is likely triggered by a lack of rooting substrate and other forms of enrichment (Henry et al. 2021). The two stages of this type are as follows: the pre-damage stage causing no visible damage, and the damaging stage in which the skin is broken (Taylor et al. 2010). The tail-in-mouth behaviour, i.e. the pre-damage stage, is considered to be normal explorative and foraging behaviour if it occurs in the majority of pigs in the population; however, it can also be a precursor and pre-injury stage of tail biting (Munsterhjelm et al. 2016). The second type, i.e. the sudden-forceful type, is described usually in standing or active pigs as grabbing and yanking the tail (Taylor et al. 2010), without the pre-injury phase and is induced by a lack of environmental resources or physical discomfort (Henry et al. 2021). The last discussed type, the obsessive one, is identified when this action is repeatedly performed, and the preliminary causes for this behaviour may be genetic (Henry et al. 2021). Both sudden-forceful and obsessive types are connected to the victim’s avoidance reaction and/or vocalisation (Taylor et al. 2010).

In general, the severity of tail-biting can range from gentle oral manipulation to actual biting of the tail, leading to serious injuries such as tail amputation, significant blood loss resulting in death, or infections affecting the whole body system (e.g., pyaemia), abscesses (mainly in the lungs), arthritis, and rump gouging (vom Brocke et al. 2019; Gomes et al. 2022). Tail bites or lesions are more visible when inspecting carcasses in slaughterhouses, and they are good indicators of poor welfare conditions in farms (van Staaveren et al. 2017). All of these issues can result in poor health status, which may require vet intervention, lead to low quality of meat products, reduced weight gain, and even death of the affected individual. In summary, these problems can cause economic losses in both farms and slaughterhouses (Telkanranta et al. 2014; Henry et al. 2021; Gomes et al. 2022; Amatucci et al. 2023).

The prevention of tail biting includes the removal of the biting animals, implementing technologies, providing access to resources, and enriching the environment and pens (Niemi et al. 2021). For example, video recording can be a helpful tool, but the initial costs and time-consuming monitoring might be a burden for breeders (Honeck et al. 2019). Thus, all these measures may be considered more expensive than the subsequent dealing with the consequences of tail biting and may be neglected by a producer or handled by tail docking instead (Taylor et al. 2010; Niemi et al. 2021). Tail docking is, however, regulated in the European Union (EU) by the EU Council Directive 2008/120/EC (EU Council 2008).

## TAIL DOCKING

Tail docking is a procedure in which a portion of the tail is removed within the first days of an animal’s life. The purpose of this procedure is to prevent tail biting, although it does not completely eliminate this problem (De Briyne et al. 2018).

This procedure can be performed as a routine preventive measure in the countries of the EU only if tail biting occurs in the herd and all other measures to reduce tail biting have been unsuccessful (Henry et al. 2021). The implementation of this procedure is subject to EU regulation.

EU Council Directive 2008/120/EC (EU Council 2008) states: “Neither tail-docking nor reduction of corner teeth must be carried out routinely but only where there is evidence that injuries to sows’ teats or to other pigs’ ears or tails have occurred. Before carrying out these procedures, other measures shall be taken to prevent tail biting and other vices, taking into account the environment and stocking densities. For this reason, inadequate environmental conditions or management systems must be changed” (Annex I, Chapter I, Point 8).

In addition to the ban on routine tail docking, European legislation requires that pigs must have “permanent access to a sufficient quantity of material to enable proper investigation and manipulation activities, such as straw, hay, wood, sawdust, mushroom compost, peat or a mixture of such” (EU Council 2008).

Although this European regulation entered into force as early as 2008, tail docking is still used by up to 95% of farms in Europe (Kakanis et al. 2021).

The mechanism by which the shortening of the tail reduces tail biting is not entirely clear. Simonsen et al. (1991) and Kakanis et al. (2021) assume that the formation of neuromas in the tail tip could lead to hypersensitivity. The same authors also propose that the hairy intact tail remains more attractive for biting.

It was found that the length of tail shortening can influence the effectiveness of this measure. According to Thodberg et al. (2018) and Kakanis et al. (2021), leaving very short tails can be more effective compared not only to undocked tails but also to tails with a longer remnant.

According to Larsen et al. (2018), tail docking in pigs has the potential to decrease tail biting 2-4-fold.

While this measure has a relatively significant potential to reduce the incidence of tail-biting in pigs, it does not completely eliminate the problem. This applies particularly to situations where predisposing factors are not addressed (EFSA 2007).

The results obtained by monitoring the incidence of tail biting on farms and at slaughterhouses show that 30% to 70% of farms in various European countries experience tail biting problems despite practicing tail docking (EFSA 2007).

Tail docking is used as a method to improve the welfare level of pigs on farms, as tail biting can negatively impact their welfare (Gerster et al. 2022).

On the other hand, the act of tail docking itself can be considered a welfare problem for pigs to some extent because it causes pain to the animals and may lead to the formation of abscesses (De Briyne et al. 2018).

It also causes stress to the pig. The stress reaction of pigs to tail docking was assessed by Morrison and Hemsworth (2020). They found that in comparison to the sham handling treatment (handling alone), tail docking increased the duration of vocalisations, the number of escape attempts during treatment, and the cortisol response at 15 min post-treatment.

Moreover, the tail condition can be used as an indicator of pig welfare status. If the tail is shortened, its use as a welfare measurement instrument is limited (Schroder-Petersen et al. 2004; Gerster et al. 2022).

## NEGATIVE EFFECTS OF PIG TAIL BITING

Tail biting is a major welfare problem for pigs because it causes pain to the affected animals. It also indicates welfare issues in pigs that engage in this type of behaviour, which often stems from abnormal behaviour due to discomfort caused by environmental inadequacies (Thodberg et al. 2018; Kakanis et al. 2021). Tail biting can cause significant economic losses to farmers, primarily resulting from reduced weight gain in the affected pigs. According to Sinisalo et al. (2012), the indirect losses due to lower average daily gain (ADG) can amount to 3%. An additional economic loss can be attributed to the increased costs of veterinary activities (Zonderland et al. 2011).

Tail biting can lead to an infection that can spread throughout the body, causing the formation of abscesses, arthritis, lung lesions, and pleurisy (Valros et al. 2004; vom Brocke et al. 2019). Therefore, a significant factor contributing to the losses could be the partial or total condemnation of slaughtered carcasses due to secondary bacterial infections (Kritas and Morrison 2007; Taylor et al. 2010; Henry et al. 2021).

## EXAMINATION OF TAIL LESIONS OF PIGS IN SLAUGHTERHOUSES

### Advantages of examination of tail lesions in slaughterhouses

Currently, there is growing interest in utilising inspections at slaughterhouses as a means of evaluating the welfare of pigs (Harley et al. 2012). Evaluating animal welfare in slaughterhouses does offer several advantages compared to traditional farm-based checks (Carroll et al. 2016). The advantage of conducting evaluations at slaughterhouses is that it eliminates problems related to compliance with biosecurity issues during farm visits. In addition, it can help avoid potential problems associated with having to evaluate animals in crowded, dirty, or poorly lit conditions (Edwards et al. 1997; Velarde et al. 2005; Carroll et al. 2016).

Many authors consider the evaluation of tail lesions in pigs at the slaughterhouse to be a good indicator for assessing the welfare state of pigs (Harley et al. 2014; van Staaveren et al. 2017). Lesions in the tail are indicative of behavioural disorders related to tail biting and reflect impaired welfare. It often points to the disharmony between the pig and its environment (Smulders et al. 2006; van Staaveren et al. 2017).

According to studies conducted by Carroll et al. (2016) and van Staaveren et al. (2017), tail lesions, especially less severe bite marks, tend to be more visible on the carcass of a slaughtered pig compared to when the pig is still alive. This represents a significant advantage of examining tail lesions at the slaughterhouse. It follows that the evaluation of tail lesions during meat inspection represents a more accurate method of the evaluation of the animal’s welfare status compared to on-farm assessments (Carroll et al. 2016; van Staaveren et al. 2017).

If a representative number of animals is examined, the examination of tail lesions of pigs at the slaughterhouse can provide important information about the welfare status of the animals on the farm (Keeling et al. 2012). Evaluating tail lesions of pigs at the slaughterhouse can also provide information about the level of the housing systems and management practices in a region. It can enable continuous animal welfare monitoring, early detection of problems, and intervention at the farm level (Gerster et al. 2022).

### Drawbacks of examination of tail lesions in slaughterhouses

The examination of pig tails at the slaughterhouse can be an important indicator of the health status of the pigs, but it applies only to those pigs that reach the slaughterhouse. When tail lesions are examined at the slaughterhouse, it does not account for pigs with severely injured tails that died due to severe lesions or were euthanised on the farm. Additionally, it also does not capture pigs with lesions that healed before reaching the slaughterhouse (Taylor et al. 2010; Marques et al. 2012; Lahrmann et al. 2017).

According to Munsterhjelm et al. (2013) and Kakanis et al. (2021), even if there are no visible external lesions on the tail, there could still be hidden bruises or more severe histopathological reactions present. Thus, tail appearance is not always the best way to quantify pigs’ tail biting activity.

A problem when monitoring tail lesions in slaughterhouses can be the fact that the observer may only record more serious lesions and miss lesions of a mild nature. This may lead to an underestimation of the actual prevalence of these lesions in slaughterhouses. In addition to the previous points, it is worth noting that healed tails are rarely recorded, and they may be indistinguishable from tail shortening (Taylor et al. 2010; Wallgren et al. 2019; Kakanis et al. 2021).

Therefore, if the goal is to evaluate the occurrence of tail biting on the farm, it is essential to carefully evaluate the findings from slaughterhouses (Lahrmann et al. 2017).

A certain underestimation can also occur due to the rapid processing of slaughtered carcasses (Kakanis et al. 2021).

The results obtained from examining tails at the slaughterhouse can indeed provide insights into the relationship between tail findings and various factors such as sex, season, and other carcass damage (Widowski 2002). However, these data may be of limited value in identifying all the factors involved in tail biting on the farm (Taylor et al. 2010; Kakanis et al. 2021).

Monitoring the level of welfare of pigs on the farm through tail lesion examination at the slaughterhouse can be quite laborious. The veterinarian’s role on the farm in analysing risk factors and developing preventive programmes remains essential (Gerster et al. 2022).

Therefore, it is important to record the prevalence of tail biting directly on the farm (Taylor et al. 2010). By documenting the prevalence of tail biting both in slaughterhouses and directly on the farm, a more comprehensive and accurate assessment of the situation can be made.

Currently, there is no uniform methodology for the evaluation of tail lesions, established for assessing tail lesions directly on the farm. The publication by Honeck et al. (2019) provides an overview of the various methods available for evaluating tail lesions directly on the farm.

### Methodical procedure in the evaluation of tail lesions in slaughterhouses

In the professional literature, there are different opinions on whether it is better to evaluate lesions on the tails of pigs before or after scalding. For example, Taylor et al. (2010) state that when inspecting carcasses after scalding, the marks left by this process may obscure the existing marks from tail injuries.

Swiss authors (Gerster et al. 2022) also recommend carrying out the evaluation of tail lesions immediately after bleeding but before the first washing and further processing of the carcasses. In their view, this rules out the possibility of tissue damage by mechanical procedures during slaughter.

The conclusions of these authors appear to be based on their personal experience and are not supported by objective data.

Recent research studies objectively evaluating this problem show that it is better to carry out the evaluation of tail lesions after scalding. For example, according to Carroll et al. (2016), tail lesions are more visible on pig carcasses after the scalding and dehairing process, making this stage the appropriate time for abattoir-based lesion scoring. They found that tail lesions of each severity category become more visible after scalding and dehairing, with a particularly high increase in visibility observed in the case of mild tail lesions. These authors also admit the possibility that damage caused to the carcass by the scalding and dehairing processes could have been misinterpreted for tail biting injuries. However, in their opinion, damages caused by technological processing can be easily distinguished from the lesions caused by tail biting and they provide the following rationale. The machinery-related damage to the carcass is manifested as shredding and peeling of the skin. These lesions lack colour, indicating that they occurred after exsanguination.

On the other hand, the lesions resulting from tail biting are coloured and exhibit bite marks. In the case of healed tail lesions, significant scar tissue may be observed.

Similar conclusions were reached by Valros et al. (2020), who also stated that it is difficult to score tails consistently before and after scalding due to the considerable difference in the appearance of the tail. Before scalding, tails are typically covered with hair, and they may be very dirty. Moreover, scabs covering the end of the tail make it difficult to assess the underlying lesions.

The main advantage of the above-mentioned two studies is the fact that the authors compared two different technological procedures used at the same time in the same study, i.e. the evaluation of tail lesions before scalding and their evaluation after scalding.

Regarding the evaluation of tail lesions, various scoring systems have been proposed, but none of them have been universally used.

Scoring systems that can be used to evaluate tail lesions in pigs can be categorised into two groups. On the one hand, there are systems that can be applicable to both docked and undocked tails, and on the other hand, there are systems that are exclusively designed for undocked tails.

### Scoring systems for docked and undocked tails

Scoring systems vary greatly between authors. For this reason, we present the scoring systems recommended by the EU.

Here is the method for the assessment of lesions on the tail of pigs, which is available on the website of the EU Reference Centre for Animal Welfare (EURCAW 2020, https://edepot.wur.nl/513891).

#### INTACT TAILS

No sign of damage: The tail is not wounded nor shortened. The tail is curled and the tail tip is flat and has bristles.

#### MINOR WOUND

Damage through the skin of at least 0.5 cm in diameter but not greater than 2.0 cm, i.e. a wound not bigger than the size of a ten-cent (euro) coin.

The wound should have fresh blood (a fresh wound), a scab (a recent but healing wound), or both.

Anything less than 0.5 cm is not scored.

#### MAJOR WOUND

Damage through the skin of at least 2.0 cm in diameter (the size of a ten-cent euro coin) or more than one minor wound (see previous definition). The wound should have fresh blood (a fresh wound), a scab (a recent but healing wound), or both.

Hereafter, we present the scoring system, which is given in a Commission Staff Working Document (EU Commission 2016b) on best practices with a view to the prevention of routine tail-docking and the provision of enrichment materials to pigs.

It accompanies the document Commission Rec-ommendation (EU Commission 2016a) on the application of Council Directive 2008/120/EC (EU Council 2008) laying down minimum standards for the protection of pigs as regards measures to reduce the need for tail-docking.

Score 1 – No evidence of tail-biting.

Score 2 – Indication of superficial biting along the length of the tail, but no evidence of fresh blood or of any swelling (red areas on the tail are not considered wounds unless associated with fresh blood).

Score 3 – Fresh blood is visible on the tail and/or there is evidence of some swelling and infection and/or part of the tail tissue is missing and a crust has formed.

### Scoring systems for undocked tails

The advantage of evaluating undocked tails is the ability to use the tail length as an important parameter. Finnish authors Valros et al. (2020) propose, based on their study’s findings, that a scoring system for tail lesions in undocked pigs should utilise both lesion scoring and tail length measurement. They also found that tails with more severe lesions and shorter lengths increased the risk for meat inspection findings. The authors use the term “intact tail”, referring to a tail that does not have substantial damage, indicating that the risk for meat inspection findings is not significantly increased (Valros et al. 2020). Defining precisely what constitutes an “intact tail” becomes crucial. These authors suggest that the definition of an intact enough tail might include tails with mild lesions (such as bite marks or bruises) and tails scored as healed, with no fresh lesions, and having more than 75% of the average intact length remaining. Such tails were found not to raise concerns for meat hygiene. A detailed description of the scoring method used by these authors is available in the methodology section of this publication.

Here we present just the evaluation method used by the authors to evaluate major and minor tail injuries.

Acute lesion, minor wound (after scalding): The tail has missing tissue, which has not fully healed yet; uneven “dents” in the skin; or a part of the tail is missing. Wound is > 0, but < 2 cm in diameter or length.

Acute lesion, major wound (after scalding): The tail has missing tissue, which has not fully healed yet; uneven “dents” in the skin; or a part of the tail is missing. The wound is 2 cm or larger in diameter or length.

Another example of the use of tail length as an important parameter for evaluating undocked tail lesions comes from the research conducted by Swiss authors (Gerster et al. 2022). They used a tail length score to facilitate better comparisons of tail lengths and a tail tip condition score. The positive outcomes from both of these studies are promising for the advancement of automatic recording systems in the future, as both tail lesions and length can be recorded by camera-based systems.

### Tail lesions and meat inspection findings

Tail biting can also be a problem for slaughterhouses because it can cause carcass lesions as a result of secondary bacterial infections. Infections originating in the tail can spread to other body regions via the bloodstream and cerebrospinal fluid (Huey 1996), potentially resulting in secondary abscessation. For instance, tails with biting lesions were found to be the source of infection for 19.9 percent of carcasses with a single abscess and 61.7 percent of carcasses with more than one abscess (Huey 1996). These infections can be caused by bacteria such as *Arcanobacter pyogenes* (akt. *Trueperella pyogenes*) and haemolytic streptococci (van den Berg et al. 1981). The authors state that they usually found more abscesses in at least one location in pigs with tail lesions (Kritas and Morrison 2007; Marques et al. 2012).

In addition, arthritis, leg inflammation, lung lesions, and pleurisy were found to be more common in pigs with tail-biting lesions (Elbers et al. 1992; Valros et al. 2004). These facts were also confirmed in a recent study by German authors (vom Brocke et al. 2019) who found that leg inflammation, arthritis, and abscesses were more prevalent in pigs withtail lesions of any degree compared to pigs without tail lesions. They also observed an association between severe tail lesions in pigs and a higher occurrence of lung lesions.

In a recent study carried out on pigs with undocked tails, Finnish authors (Valros et al. 2020) demonstrated that as a larger portion of the tail was missing, the level of secondary infections increased. It resulted in higher carcass condemnations in pigs with a large proportion of the tail missing.

In general, it can be concluded that more severe tail lesions are more closely connected to carcass trimming than milder lesions (Kritas and Morrison 2007). Nonetheless, even mild tail lesions can lead to carcass condemnations (Harley et al. 2014).

Therefore, tail biting can cause economic losses due to partial or full carcass condemnations resulting from secondary infections associated with tail lesions (Huey 1996; Kritas and Morrison 2007). Harley et al. (2014) estimated that partial or full carcass condemnations can reduce the economic gain by € 1.10 per pig. Another cause of indirect financial losses for breeders can be the fact that slaughtered carcasses of pigs affected by tail biting tend to have a lower weight (Harley et al. 2014). The explanation may be the fact that tail lesions can cause reduced feed intake and growth due to the effects of infection and stress (Marques et al. 2012).

### Tail lesion prevalence

Here we present examples of the detected prevalence of tail lesions in pigs as found in slaughterhouses in Europe. Estimates of tail damage prevalence from most countries are based on specific studies.

#### UNITED KINGDOM

Hunter et al. (1999) reported that 9% of undocked pigs and 3% of docked pigs had damaged tails. They also found that 0.1% of docked and 0.5% of undocked pigs had the most severe form of damage.

#### FINLAND

Valros et al. (2004) reported that the total prevalence of tail damage in pigs with undocked tails was 34.6% and that the prevalence of tails with severe damage was 1.3%.

#### SWEDEN

In a study conducted by Keeling et al. (2012), tail damage and tail length in pigs with undocked tails were recorded at two slaughterhouses in Sweden. The total prevalence of injury and of tail shortening was 7.0% and 7.2% in slaughterhouses A and B, respectively. When only considering pigs with half or less of the tail left, these percentages were 1.5% and 1.9%.

#### GERMANY

In a study by vom Brocke et al. (2019), the mean batch prevalence for tail lesions in pigs with docked tails was 25.4% when considering all lesions and 1.88% when only severe lesions were included.

#### SWITZERLAND

In a study by Gerster et al. (2022), lesions of undocked tails were evaluated. Overall, 63.2% of the animals included in the analysis were slaughtered with a complete tail, whereas 36.8% showed a partial or total loss of the tail. The condition of the tail tip was judged as intact in 63.0%, showing a healed lesion in 23.7%, an acute lesion in 1.3%, and a chronic lesion in 12.0%.

#### PORTUGAL

In a study by Franco et al. (2021), lesions of docked tails were evaluated. They found that 84% pig carcasses showed no tail lesions. Mild to moderate tail lesions were found in 13% and severe ones in 3% of pigs.

#### REPUBLIC OF IRELAND

In a study by Harley et al. (2014), lesions of docked tails were evaluated. Tail lesions were observed in 72.5% of the pigs under study, with 2.5% affected by severe tail lesions.

From the given examples, it is clear that the prevalence of tail lesions found in slaughterhouses shows a very high variability between studies. Therefore, data from different countries are difficult to compare. The following factors can be cited as possible causes of such a high variability.

Tail biting in pigs is a multifactorial problem, leading to significant variations in the prevalence of lesions among pig herds (Moinard et al. 2003).

Another cause of high variability may be the fact that different systems of lesion grading were used in individual studies (Keeling et al. 2012; Harley et al. 2014). A difficult situation can arise in the evaluation of mild lesions because the scoring of their severity can be influenced by the investigators themselves, i.e. by differences in estimation between individuals (Keeling et al. 2012).

It can also be influenced by the phase of processing of the carcasses during which the evaluation is carried out (Carroll et al. 2016), i.e., whether the evaluation of tail lesions was performed before or after the carcasses were scalded.

## SUMMARY

Pig tail biting poses a serious challenge in current pig farming, with numerous contributing factors. While many risk factors are known, identifying the exact cause on a specific farm proves challenging in practice. Monitoring this problem through slaughterhouse inspections presents a viable option. Among the main advantages of such an approach is the fact that tail lesions are more visible on the carcass, especially less severe bite marks, compared to live animals.

However, it requires significant labour and demands a farm-specific analysis to identify triggering factors accurately. Nonetheless, conducting examinations of pig tail lesions at slaughterhouses using a representative sample can provide valuable insights into the welfare status of the animals on the farm, thereby enabling early detection and intervention.
